# Editorial: Deep Eutectic Solvents/Complex Salts-Based Electrolyte for Next Generation Rechargeable Batteries

**DOI:** 10.3389/fchem.2020.613353

**Published:** 2020-11-26

**Authors:** Du Yuan, Gen Chen, Chuankun Jia, Haitao Zhang

**Affiliations:** ^1^School of Materials Science and Engineering, Nanyang Technological University, Singapore, Singapore; ^2^School of Materials Science and Engineering, Central South University, Changsha, China; ^3^School of Materials Science and Engineering, Changsha University of Science and Technology, Changsha, China; ^4^Key Laboratory of Green Process and Engineering, Institute of Process Engineering, Chinese Academy of Sciences, Beijing, China

**Keywords:** SEI, redox, electrochemisitry, rechargeable batteries, electrolyte

Recent years have seen an expansion of renewable energy technologies driven by global demands for energy alongside social and environmental concerns. One of the most significant solutions, rechargeable batteries have promising features which include high capacity, energy density, rate capability, long lifetime, and cost-effectiveness. As the key component in energy storage devices, the electrolyte has had a major impact on the chemistry/electrochemistry of rechargeable batteries/cells for a number of reasons. These include its potential window, which limits the redox potential of an electrochemical reaction. Its electrochemical activity and conductivity also influence the electrochemical reaction and consequently the battery performance. The composition, as well as the stability, of rechargeable batteries, shapes the electrolyte-electrode interface. Furthermore, its corrosivity cannot be neglected. For these reasons, researchers are highly motivated toward breakthroughs in battery performance, exploring the fundamental properties of electrolytes based on novel formulation/synthesis. Hence, this special issue of *Deep Eutectic Solvents/Complex Salts-Based Electrolyte for Next Generation Rechargeable Batteries* focuses on the effects of electrolytes on the electrochemistry/chemistry of rechargeable batteries and cells.

In this Research Topic, representative types of electrolytes are discussed in relation to next-generation rechargeable batteries, including ionic liquid, solid state electrolyte (polymeric and inorganic), multivalent electrolyte (aqueous and non-aqueous) for multivalent ion batteries, aqueous, and a novel organic electroactive molecule-based electrolyte for flow batteries. Recent developments build upon the correlation between design and synthesis of the electrolyte, its properties, and the related electrochemical properties and performance of batteries and cells. From the perspective of electrolyte design, contributions include exploration of formulating ionic liquid toward designed ion separation for tuning the performance of supercapacitors (Pan et al.). Other submissions discuss the compositing strategy for a polyethylene oxide-based solid state electrolyte for flexible device and lithium metal batteries (Zhao et al.). Another study considers the emerging ionic conductor for an Mg ion solid state electrolyte (Zhan et al.), where another describes how electrolyte modification can be used to depress zinc dendrite in zinc-based flow batteries (Guo et al.). The electrolyte concentration, as well as cation/proton types of diffusion kinetics in a vanadium flow battery (Gui et al.) are also interest. Another contribution examines the molecular design of organic electroactive molecules via functional group modification for redox flow batteries (Zhong et al.). Furthermore, the electrolyte-electrode interface is also emphasized in this topic, where its material characteristics and stability strongly influence the kinetics and cycling stability of batteries/cells for example in an *in situ* Scanning Electrochemical Microscopy study, which revealed that the SEI formation on carbon depends on voltage range (Liu et al.), or, as described elsewhere, regulating the nature of ionic liquid and the interface compatibility (Pan et al.). Another study designed a novel 3D MoSe_2_ template for constructing stable SEI for reversible stripping/plating of metallic Mg (Shen et al.), where others discuss regulating the interface between electrolyte and Zn for preventing zinc dendrites (Guo et al.), or investigating the interfacial charge transfer in aqueous zinc electrolytes for zinc ion batteries (Guo et al.).

We would like to thank all the authors for their valuable contributions and the reviewers for their thoughtful insights and suggestions. It is essential to further examine and share the fundamentals of the chemistry and electrochemistry of electrolytes, which serves as the basis for the successful creation of next-generation rechargeable energy storage devices.

Although the papers included here provide significant insights, there are still important developments and points for the future development of this area of research. Safety is a long-term pursuit in rechargeable batteries, and further insights into safe electrolyte design with promising electrochemical properties are essential. The pursuit of high performance and long lifetime in rechargeable batteries will also need to go further than constructing a database of electrolytes, and future explorations on the relation between electrolytes and battery chemistry are essential. It would also be promising to uncover the correlation between electrolyte and charge storage mechanism for any novel electrodes designs, which will synergistically guide the development of new generation battery systems. Moreover, the complexity of the electrolyte-electrode interface creates room for exploring battery chemistry, which will drive cutting-edge characterization and analysis tools and in parallel, boost the development of new battery systems, especially those for utilizing metal anode based or multivalent ion batteries.

We hope that this special issue will inspire future research in the development of novel electrolytes and drive the development of new generation rechargeable energy storage devices. These endeavors will pave the way for realizing a green and sustainable society.

## Author Contributions

DY, GC, CJ, and HZ co-edited this special issue. All authors contributed to the article and approved the final manuscript.

## Conflict of Interest

The authors declare that the research was conducted in the absence of any commercial or financial relationships that could be construed as a potential conflict of interest.

